# The Role of Galectin-3 in 1α,25(OH)_2_D_3_-Regulated Osteoclast Formation from White Leghorn Chickens In Vitro

**DOI:** 10.3390/vetsci8100234

**Published:** 2021-10-14

**Authors:** Jianhong Gu, Wenyan Min, Yutian Zhao, Xueqing Zhang, Yan Yuan, Xuezhong Liu, Jianchun Bian, Xishuai Tong, Zongping Liu

**Affiliations:** 1 College of Veterinary Medicine, Yangzhou University, Yangzhou 225009, China; jhgu@yzu.edu.cn (J.G.); MX120170751@yzu.edu.cn (W.M.); ytzhao202109@163.com (Y.Z.); MX120190776@yzu.edu.cn (X.Z.); yuanyan@yzu.edu.cn (Y.Y.); xzliu@yzu.edu.cn (X.L.); jcbian@yzu.edu.cn (J.B.); 2 Jiangsu Co-Innovation Center for Prevention and Control of Important Animal Infectious Diseases and Zoonoses, Yangzhou 225009, China; 3 Jiangsu Key Laboratory of Zoonosis, Yangzhou 225009, China; 4 Institutes of Agricultural Science and Technology Development (Joint International Research Laboratory of Agriculture and Agri-Product Safety of the Ministry of Education of China), Yangzhou University, Yangzhou 225009, China

**Keywords:** galectin-3 (Gal-3), bone marrow stromal cells (BMSCs), bone marrow monocytes/macrophages (BMMs), osteoclasts (OCs), 1α,25-dihydroxyvitamin D_3_ (1α,25(OH)_2_D_3_)

## Abstract

Bones play an important role in maintaining the level of calcium in blood. They provide support for soft tissues and hematopoiesis and undergo continuous renewal throughout life. In addition, vitamin D is involved in regulating bone and calcium homeostasis. Galectin-3 (Gal-3) is a β-galactoside-binding protein that can regulate bone cell differentiation and function. Here, we aimed to study the regulatory effects of Gal-3 on vitamin-D-regulated osteoclastogenesis and bone resorption in chicken. Gal-3 expression in bone marrow stromal cells (BMSCs) from 18-day-old chicken embryos was inhibited or overexpressed. BMSCs were then co-cultured with bone marrow monocytes/macrophages (BMMs) with or without addition of 1α,25(OH)_2_D_3_. The results showed that 1α,25(OH)_2_D_3_ upregulated the expression of *Gal-3* mRNA and receptor activator of nuclear-factor κB ligand (*RANKL*) expression in BMSCs and promoted osteoclastogenesis, as shown by the upregulated expression of osteoclast (OC) markers (*CtsK*, *CAII*, *MMP-9*, and *TRAP*) and increased bone resorption, a method for measuring the bone resorption area in vitro. Knockdown of *Gal-3* by small-interfering RNA (siRNA) in BMSCs downregulated the expression of *RANKL* mRNA and attenuated the effects of 1α,25(OH)_2_D_3_ on osteoclastogenesis and bone resorption. Conversely, overexpression of *Gal-3* in BMSCs enhanced the effects of osteoclastogenesis and bone resorption by increasing the expression of *RANKL* mRNA. These results demonstrated that Gal-3 mediates the differentiation and bone resorption of osteoclasts regulated by 1α,25(OH)_2_D_3_.

## 1. Introduction

Bone homeostasis is achieved by osteogenesis and bone resorption, which are regulated by osteoblasts (OBs) and osteoclasts (OCs), respectively [1,2]. OCs are specifically responsible for physiological and pathological bone resorption. Excessive increase or decrease in their quantity and activity is detrimental to bone and calcium (Ca) homeostasis [3,4]. Vitamin D is a sterol derivative and has long been considered to promote osteogenesis by promoting the intestinal transport of Ca and osteolysis, ensuring the stability of serum calcium homeostasis. Previous studies have shown that 1α,25-dihydroxyvitamin D_3_ (1α,25[OH]_2_D_3_) can upregulate receptor activator of nuclear-factor κB ligand (RANKL) expression in OBs and bone marrow stromal cells (BMSCs) in mammalians, indirectly inducing bone marrow monocytes/macrophages (BMMs) to differentiate into OCs for bone resorption [5,6,7].

Galectin-3 (Gal-3) is a member of the β-galectin family and presents in the nucleus. Gal-3 can regulate cell migration, adhesion, apoptosis, and gene expression [8,9]. Studies have shown that Gal-3 is expressed in chondrocytes, OBs, BMSCs, and OCs and regulates osteocyte differentiation and function [10,11]. In *Gal-3* (*Gal-3*^−/−^) knockout mice, OB and OC differentiation and bone resorption were impaired [12]. It has also been confirmed that Gal-3 could overlap with the transcription factor Runx2, which regulates OB differentiation [13]. Furthermore, Gal-3 can regulate bone marrow mesenchymal stem cell migration through RhoA-GTP signaling and may be a potential target for treating bone reconstruction-related diseases [14]. Aubin et al. found that 1α,25(OH)_2_D_3_ promoted Gal-3 expression in a rat OB-derived sarcoma cell line, ROS 17/2.8 [15]. Simon et al. reported that knockout *Gal-3* in OBs displayed higher osteoclastogenesis, independently of the RANKL signaling pathway [16]. However, the role of Gal-3 in 1α,25(OH)_2_D_3_-regulated osteoclastogenesis and bone resorption and whether it affects vitamin D regulation of osteoclastogenesis in poultry (chicken) is unclear.

Chicken bones have been used in bone development and bone injury studies for a long time as they are similar to those of humans and other vertebrates, and vitamin D has been found to regulate bone development in chickens [17,18,19]. Our previous study showed that 1α,25(OH)_2_D_3_ promoted osteoclastogenesis in a chicken BMSC–BMM co-culture system in a dose-dependent manner, with 10^−8^ mol/L having the most significant effect [20]. In addition, 10^−8^ mol/L 1α,25(OH)_2_D_3_ was used in the current study to further examine the effects of vitamin D on Gal-3 expression in BMSCs, BMMs, and BMSC–BMM co-culture. Small-interfering RNA (siRNA) and gene overexpression were used to knockdown or overexpress *Gal-3* to observe the effects of 1α,25(OH)_2_D_3_ on osteoclastogenesis, bone resorption, and RANKL signaling and to examine whether Gal-3 affects 1α,25(OH)_2_D_3_ regulation of osteoclastogenesis through RANKL signaling. These results will provide a foundation for studies on vitamin D regulation of bone and calcium homeostasis in poultry.

## 2. Materials and Methods

### 2.1. Animals

The white leghorn chicken embryos used here were fertilized SPF-grade eggs from Single Comb White Leghorn (Yigida Biotechnology, Jining, China). The SPF-grade eggs were incubated at 37 °C and 60% humidity until they were 18 days old. The animal use was approved by the Animal Care and Use Committee of Yangzhou University (SYXK [Su] 2016-0020).

### 2.2. Isolation of BMSCs and BMMs

Tibias and femurs were separated, and bone marrow cells were filtered with a 200-mesh sieve and then centrifuged at 1200 r/min for 5 min. The cells were subsequently resuspended in α-MEM (Thermo Fisher Scientific, Waltham, MA, USA) supplemented with 10% FBS (EallBio, Beijing, China) and then incubated in an incubator (5% CO_2_, 37 °C). After 2 days, the BMSCs were adherent and the BMMs were non-adherent.

### 2.3. Transfection

The sequence of *Gal-3* siRNA was designed as 5′-AGAGAACAGCTCCTAGATT-3′, and the sequences of negative control (NC) siRNA were designed as 5′-GGCTCTAGAAAAGCCTATGC-3′ (RiboBio, Guangzhou, China).

BMSCs were seeded in cell cultural plates (Corning, New York, NY, USA) or bone resorption cultural plates (Sigma-Aldrich, St. Louis, MO, USA). After 12 h, the BMSCs were transfected with siRNAs. Transfection was performed using Lipofectamine^TM^ 3000 (Thermo Fisher Scientific, Waltham, MA, USA) according to the manufacturer’s instructions, as previously reported [21].

### 2.4. Overexpression of Gal-3 Plasmids

Gal-3 homologous recombination was performed with PEXP-RB-MAM-EGFP transient vector (RiboBio, Guangzhou, China). The gene EGFP-Gal-3 was used for cell transfection, and the empty carrier control was named EGFP. Transfection was performed using Lipofectamine^TM^ 3000 (Thermo Fisher Scientific, Waltham, MA, USA) according to the manufacturer’s instructions, as previously reported [21].

### 2.5. Co-Culture of BMSCs and BMMs

BMSCs were seeded into cell cultural plates or bone resorption cultural plates at a ratio of 1:100 (BMSCs:BMMs). Cells were then treated with 10^−8^ mol/L 1α,25(OH)_2_D_3_ (Sigma-Aldrich, USA) for 5 d (control group without 1α,25(OH)_2_D_3_). The medium was changed every 2 days. At the end of the incubation period, the medium was decanted from the cell cultures. BMSCs were transfected with NC siRNA and *Gal-3* siRNA for 10 h. BMMs were seeded into these transfected BMSC cultures at a ratio of 1:100 (BMSCs:BMMs).

At the end of the incubation period, the medium was decanted from the cell cultures. BMSCs were transfected with EGFP plasmid and EGFP-Gal-3 for 10 h. BMMs were transferred into BMSCs at a ratio of 100:1 (BMMs:BMSCs). Then, the medium was changed to α-MEM (containing 10% FBS) for 5 d. The medium was changed every 2 days.

### 2.6. Identification of Osteoclastogenesis

At the end of the incubation period, the medium was decanted from the cell cultures. Cells were fixed with 4% paraformaldehyde for 10 min (New Cell & Molecular Biotech Co., Ltd., Hangzhou, China). Tartrate-resistant Acid Phosphatase (TRAP) staining solution was added according to the manufacturer’s instructions (Sigma-Aldrich, St. Louis, MO, USA), as previously described [22].

### 2.7. Identification of Bone Resorption

At the end of the incubation period, the medium was decanted from the cell cultures. PBS was used to repeatedly wash the plates, and photographs were subsequently taken under an inverted microscope. Image-Pro Plus software was used for calculation of the area of the bone resorption pits.

### 2.8. qRT-PCR

RNA was extracted using Trizol (Thermo Fisher Scientific, Waltham, MA, USA) and qRT-PCR following the manufacturer’s instructions, as previously described [23]. The expression of the targeting genes cathepsin K (*CtsK*), *TRAP*, matrix metalloproteinase-9 (*MMP-9*), carbonic anhydrase II (*CAII)*, *RANKL*, osteoprotegrin (*OPG*), and *Gal-3* mRNA was measured. GAPDH was used as an internal reference. All primers used are shown in Table 1.

### 2.9. ELISA

The supernatants of cells were collected, and the levels of CtsK, MMP-9, RANKL, and OPG were measured using chicken ELISA kits (Shanghai Enzyme-linked Biotechnology Co., Ltd., Shanghai, China) according to the manufacturer’s instructions. The OD value was measured at 450 nm within 15 min. Protein content was calculated based on the OD.

### 2.10. Statistical Analysis

Results are expressed as the mean ± SD from at least three independent experiments. Significance was calculated by one-way analysis of variance (ANOVA) using SPSS 19.0 software. *p*-values lower than 0.05 were considered statistically significant.

## 3. Results

### 3.1. 1α,25(OH)_2_D_3_ Promoted Osteoclastogenesis and Bone Resorption

**Figure 1:**
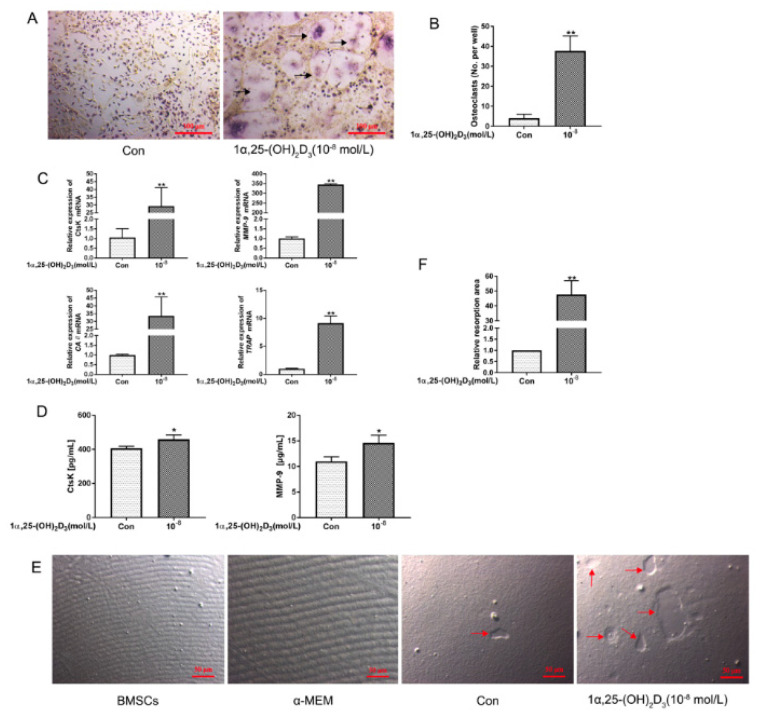
Culture with 10^−8^ mol/L 1α,25(OH)_2_D_3_ promotes osteoclastogenesis and bone resorption in the chicken BMSC–BMM co-culture system. (**A**,**B**) BMSCs and BMMs were co-cultured on 6-well plates with or without (Con) addition of 10^−8^ mol/L 1α,25(OH)_2_D_3_. Cells were treated for 5 d, and 10 fields were randomly selected. The number of TRAP-positive multinucleated OCs (black arrows) was counted. Bar = 100 μm. (**C**,**D**) BMSCs and BMMs were co-cultured on 6-well plates with or without (Con) addition of 10^−8^ mol/L 1α,25(OH)_2_D_3_. Cells were treated for 5 d, and qRT-PCR was used to quantitate the mRNA expression of OC marker genes (*CtsK*, *TRAP*, *MMP-9*, and *CAII)*. Chicken ELISA kits were used to measure CtsK and MMP-9 protein levels in the cell culture supernatant (*n* = 3 per group). (**E**,**F**) BMSCs alone, no cells, and BMSC–BMM were added to a 96-well bone resorption culture plate. After that, 10^−8^ mol/L 1α,25(OH)_2_D_3_ was added or not added (Con) to BMSC–BMM co-culture wells for 5 d. An inverted microscope was used to observe 10 randomly selected fields. Image-Pro Plus 6.0 software was used to analyze the area of bone resorption pits (red arrows). Bar = 50 μm. (Graph bars show mean ± SD. * *p* < 0.05 and ** *p* < 0.01 show significant difference compared with the control group).

BMSCs were co-cultured with BMMs for 5 d with or without the addition of 10^−8^ mol/L 1α,25(OH)_2_D_3_. As shown in Figure 1A,B, 10^−8^ mol/L 1α,25(OH)_2_D_3_ significantly (*p* < 0.01) promoted osteoclastogenesis, characterized by multinucleation and the wine-colored cytoplasm. Similarly, 10^−8^ mol/L 1α,25(OH)_2_D_3_ significantly (*p* < 0.01) upregulated the expression of *CtsK*, *TRAP*, *MMP-9*, and *CAII* mRNA (Figure 1C) and significantly (*p* < 0.05) increased the protein levels of CtsK and MMP-9 in the medium supernatant (Figure 1D).

In addition, the area of bone resorption pits was significantly (*p* < 0.01) increased in the 10^−8^ mol/L 1α,25(OH)_2_D_3_ group (Figure 1E,F). These results show that 10^−8^ mol/L 1α,25(OH)_2_D_3_ promotes osteoclastogenesis and bone resorption in the chicken BMSC–BMM co-culture system.

### 3.2. 1α,25(OH)_2_D_3_ Promoted the Expression of Gal-3

**Figure 2:**
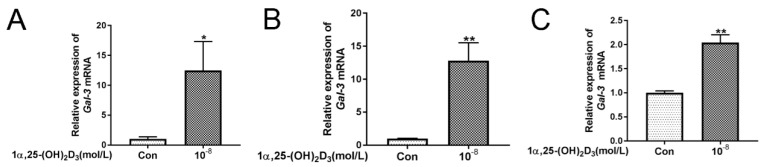
Application of 10^−8^ mol/L 1α,25(OH)_2_D_3_ upregulates the expression of *Gal-3* mRNA in the BMSC–BMM co-culture system and monocultures. (**A**) BMSCs and BMMs were co-cultured in 6-well plates with or without (Con) 1α,25(OH)_2_D_3_. Quantitative RT-PCR was used to quantitate the expression of *Gal-3* mRNA. (**B**) BMSCs were seeded in 6-well plates with or without (Con) 1α,25(OH)_2_D_3_, and qRT-PCR was used to quantitate the expression of *Gal-3* mRNA. (**C**) BMMs were seeded in 6-well plates with or without (Con) 1α,25(OH)_2_D_3_, and qRT-PCR was used to quantitate the expression of *Gal-3* mRNA. (Graph bars show mean ± SD. * *p* < 0.05 and ** *p* < 0.01 show significant difference compared with the control group; *n* = 3 per group).

Application of 10^−8^ mol/L 1α,25(OH)_2_D_3_ significantly (*p* < 0.05 or *p* < 0.01) upregulated the expression of *Gal-3* mRNA in the BMSC–BMM co-culture system, BMSCs, and BMMs (Figure 2A–C). These results indicated that 10^−8^ mol/L 1α,25(OH)_2_D_3_ increased the *Gal-3* expression.

### 3.3. 1α,25(OH)_2_D_3_ Promoted RANKL Expression and Inhibited OPG Expression

**Figure 3:**
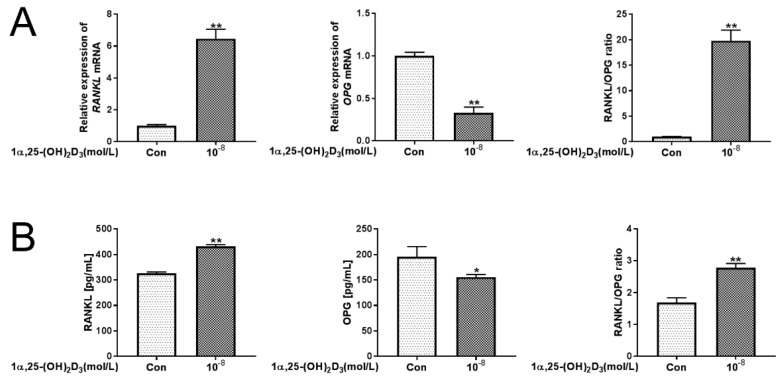
Application of 10^−8^ mol/L 1α,25(OH)_2_D_3_ upregulates the RANKL/OPG ratio in BMSCs. (**A**) BMSCs were seeded in 6-well plates with or without (Con) 1α,25(OH)_2_D_3_. After 5 d, qRT-PCR was used to quantitate the expression of *RANKL* and *OPG* mRNA, and the RANKL/OPG ratio was calculated. (**B**) BMSCs were seeded in 6-well plates with or without (Con) 1α,25(OH)_2_D_3_. After 5 d, the cell supernatant was collected, and ELISA kits were used to measure RANKL and OPG protein levels. (Graph bars show mean ± SD. * *p* < 0.05 and ** *p* < 0.01 show significant difference compared with the control group; *n* = 3 per group.).

Application of 10^−8^ mol/L 1α,25(OH)_2_D_3_ significantly (*p* < 0.01) inhibited the expression of *OPG* mRNA in BMSCs, but the expression of *RANKL* mRNA and the ratio of *RANKL*/*OPG* mRNA were significantly (*p* < 0.01) increased compared with the control group (Figure 3A). Similarly, 10^−8^ mol/L 1α,25(OH)_2_D_3_ significantly (*p* < 0.01) upregulated the protein levels of RANKL protein and the ratio of RANKL/OPG protein in the medium supernatant but downregulated the protein levels of OPG protein (Figure 3B). This shows that 10^−8^ mol/L 1α,25(OH)_2_D_3_ increased the RANKL/OPG ratio in BMSCs.

### 3.4. Knockdown of Gal-3 Inhibited Osteoclastogenesis

**Figure 4:**
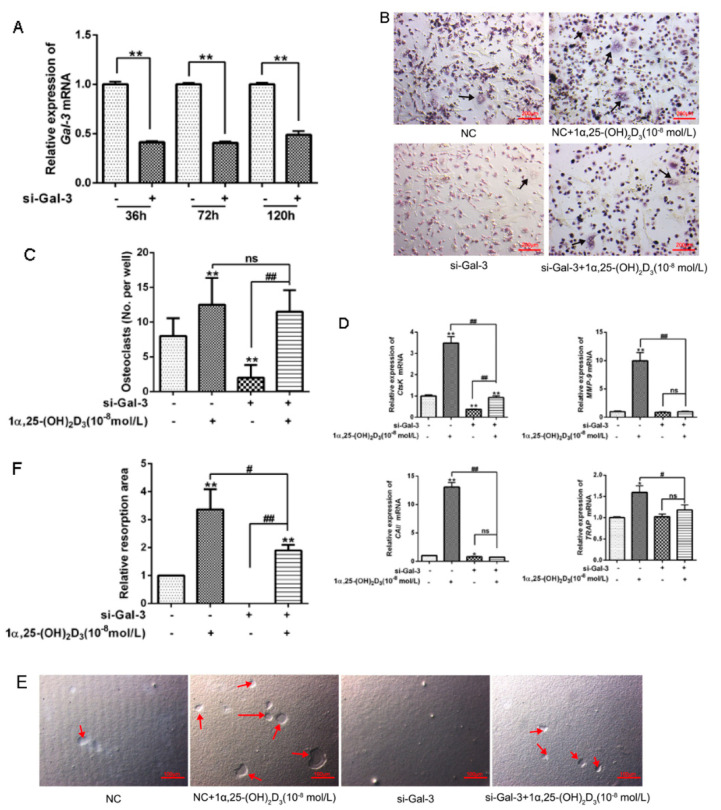
*Gal-3* knockdown in BMSCs decreases the promoting effects of 10^−8^ mol/L 1α,25(OH)_2_D_3_ on osteoclastogenesis and bone resorption. (**A**) BMSCs were transfected with si-Gal-3 or NC siRNA for 10 h before being cultured in α-MEM for 36, 72, or 120 h. Quantitative RT-PCR was used to quantitate the expression of *Gal-3* mRNA (*n* = 3 per group). (**B**,**C**) After *Gal-3* knockdown in BMSCs, BMSCs and BMMs were co-cultured on 6-well plates with or without addition of 1α,25(OH)_2_D_3_. Cells were cultured for 5 days. NC was the blank control. Ten fields were randomly selected. The number of TRAP-positive multinucleated OCs (black arrows) was counted. Bar = 200 μm. (**D**) After *Gal-3* knockdown, BMSCs were co-cultured with BMMs with or without addition of 1α,25(OH)_2_D_3_. Cells were cultured for 5 d. NC was the blank control. Quantitative RT-PCR was used to quantitate the mRNA expression of OC marker genes (*CtsK*, *CAII, MMP-9*, and *TRAP*) (*n* = 3 per group). (**E**,**F**) After BMSCs were seeded in 96-well bone resorption culture plates and *Gal-3* was knocked down, BMSCs and BMMs were co-cultured with or without addition of 1α,25(OH)_2_D_3_. Cells were cultured for 5 days. NC was the blank control. An inverted microscope was used for observation of 10 randomly selected fields. Image-Pro Plus 6.0 software was used to analyze the area of bone resorption pits (red arrows). Bar = 100 μm. (Graph bars show mean ± SD. * *p* < 0.05 and ** *p* < 0.01 show significant difference compared with the NC control group. ^#^ *p* < 0.05 and ^##^ *p* < 0.01 show significant difference compared with the 1α,25(OH)_2_D_3_-treated si-Gal-3 group).

BMSCs were transfected with targeting of Gal-3 siRNA for 36, 72, and 120 h, and the expression of *Gal-3* mRNA was significantly (*p* < 0.01) decreased in the si-Gal-3 group at each time point (Figure 4A).

As shown in Figure 4B–F, 10^−8^ mol/L 1α,25(OH)_2_D_3_ significantly (*p* < 0.01 or *p* < 0.05) promoted osteoclastogenesis compared with the NC group; upregulated the expression of *CtsK*, *TRAP*, *MMP-9*, and *CAII* mRNA; and enhanced the activity of bone resorption. However, knockdown of Gal-3 suppressed the expression of OC markers mRNA and the activity of bone resorption under treatment with 10^−8^ mol/L 1α,25(OH)_2_D_3_. These results show that 10^−8^ mol/L1α,25(OH)_2_D_3_ regulates osteoclastogenesis and bone resorption in BMSCs through the regulation of Gal-3.

### 3.5. EGFP-Gal-3 Promoted OC Differentiation

**Figure 5:**
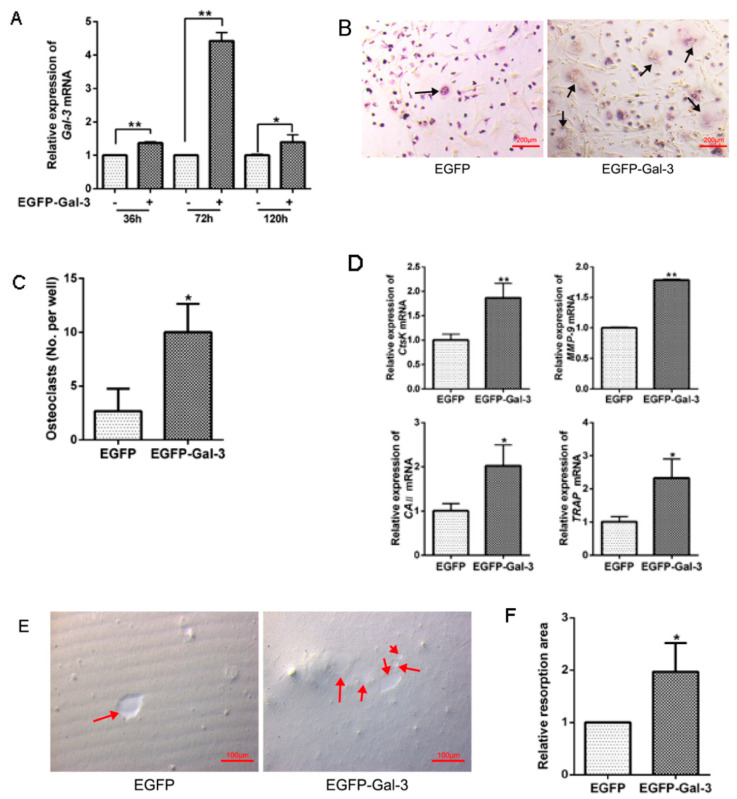
*Gal-3* overexpression in BMSCs promotes osteoclastogenesis and bone resorption. (**A**) BMSCs were seeded for 12 h before transfection with EGFP-Gal-3 or EGFP empty plasmid for 10 h before being cultured in normal α-MEM for 36, 72, or 120 h. Quantitative RT-PCR was used to quantitate the expression of *Gal-3* mRNA (*n* = 3 per group). (**B**,**C**) After *Gal-3* was overexpressed in BMSCs, BMSCs and BMMs were co-cultured for 5 d. An EGFP empty control was set up. Ten fields were randomly selected. The number of TRAP-positive multinucleated OCs (black arrows) was determined. Bar = 200 μm. (**D**) After *Gal-3* was overexpressed in BMSCs, BMSCs were co-cultured with BMMs. Quantitative RT-PCR was used to quantitate the mRNA expression of OC marker genes (*CtsK*, *CAII, MMP-9*, and *TRAP*) (*n* = 3 per group). (**E**,**F**) After BMSCs were seeded in 96-well bone resorption culture plates and *Gal-3* was overexpressed, BMSCs and BMMs were co-cultured for 5 days. An inverted microscope was used for observation of 10 randomly selected fields. Image-Pro Plus 6.0 software was used to analyze the area of bone resorption pits (red arrows). Bar = 100 μm. (Graph bars show mean ± SD. * *p* < 0.05 and ** *p* < 0.01 show significant difference compared with the control group).

BMSCs were transfected with EGFP-Gal-3 or EGFP for 36, 72, and 120 h. Compared with the EGFP group, the expression of *Gal-3* mRNA significantly (*p* < 0.01) increased following treatment with EGFP-Gal-3 (Figure 5A). In addition, the amount of TRAP; the expression of *CtsK*, *TRAP*, *MMP-9*, and *CAII* mRNA; and the area of bone resorption were significantly (*p* < 0.01 or *p* < 0.05) increased in the EGFP-Gal-3 group (Figure 5B–F). These results indicate that overexpression of Gal-3 in BMSCs could upregulate osteoclastogenesis and bone resorption.

### 3.6. Effects of Si-Gal-3 and EGFP-Gal-3 on RANKL Expression in BMSCs

**Figure 6:**
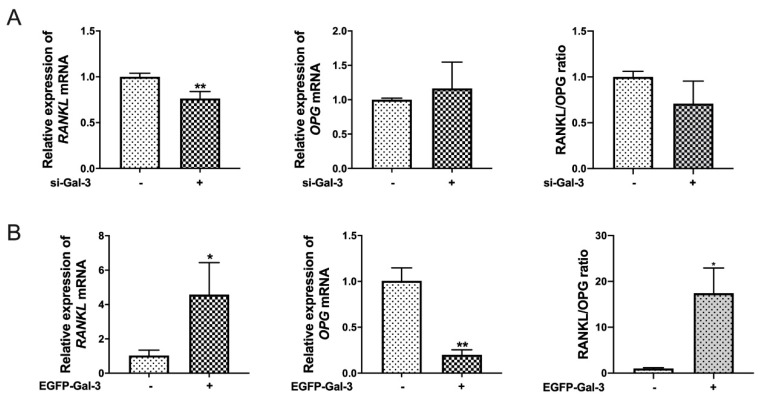
Gal-3 can regulate RANKL signals. (**A**) BMSCs were transfected with si-Gal-3 or NC siRNA for 10 h before being cultured in normal α-MEM for 120 h. Quantitative RT-PCR was used to measure *RANKL* and *OPG* mRNA levels to analyze the RANKL/OPG ratio (*n* = 3 per group). (**B**) BMSCs were transfected with EGFP-Gal-3 or EGFP empty plasmid for 10 h before being cultured in normal α-MEM for 120 h. Quantitative RT-PCR was used to measure *RANKL* and *OPG* mRNA levels to analyze the RANKL/OPG ratio (*n* = 3 per group). (Graph bars show mean ± SD. * *p* < 0.05 and ** *p* < 0.01 show significant difference compared with the control group).

As shown in Figure 6, si-Gal-3 significantly (*p* < 0.01) inhibited the expression of *RANKL* mRNA, but there was no significant (*p* > 0.05) difference in the expression of *OPG* mRNA and the ratio of *RANKL*/*OPG*. Conversely, overexpression of Gal-3 significantly (*p <* 0.05) upregulated the expression of *RANKL* mRNA and the ratio of *RANKL*/*OPG* but significantly inhibited *OPG* expression (*p* < 0.01) (Figure 6B). These results show that Gal-3 could regulate RANKL expression in BMSCs.

## 4. Discussion

Bone homeostasis depends on bone resorption in mammals and poultry; otherwise, it will lead to bone metabolism disorders. BMSCs are the progenitors of various cells (e.g., chondrocytes, adipocytes, and osteoblasts) which are recruited for microenvironmental stimulation of the bone surface, regulating osteoclastogenesis directly or indirectly [2,24]. BMSCs can secrete cytokines (e.g., RANKL and OPG) to regulate osteoclastogenesis [25,26,27]. In addition, activation of vitamin D promotes RANKL expression in BMSCs and OBs in vitro, regulating osteoclastogenesis [6,7,28]. In this study, treatment with 10^−8^ mol/L 1α,25(OH)_2_D_3_ promoted the expression of RANKL and osteoclastogenesis.

Gal-3 is expressed at different locations in cells, including the nucleus, extracellularly, and in the cell membrane. Ortega et al. showed that Gal-3, an in vitro substrate of MMP-9, accumulates in late hypertrophic chondrocytes and the expanded hypertrophic cartilage zone. Furthermore, treatment with full-length Gal-3, but not that cleaved by MMP-9, led to expansion of the hypertrophic zone and inhibited osteoclast recruitment [29]. However, Nakajima et al. showed that Gal-3 cleavage in the bone tumor microenvironment (TME) of breast and prostate cancers delays osteoclastogenesis by targeting the carbohydrate recognition domain (CRD) of Gal-3 [30]. Next, we aimed to observe the role of Gal-3 in osteoclastogenesis in BMSCs and BMM co-culture system treated by 1α,25(OH)_2_D_3_. We found that application of 10^−8^ mol/L 1α,25(OH)_2_D_3_ enhanced the expression of *Gal-3* in BMSCs. A previous study reported that 1α,25(OH)_2_D_3_ upregulated the expression of Gal-3 in OBs [15]. In addition, knockout of Gal-3 in OBs and/or BMMs in mice caused osteoclastogenesis, indicating that Gal-3 is involved in the differentiation of osteoclasts [16]. Our research found that knockdown of Gal-3 in BMSCs co-cultured with BMMs could suppress osteoclastogenesis and bone resorption. However, overexpression of Gal-3 could reverse it. In this study, we did not investigate the role of Gal-3 in regulating RANK in monocyte/macrophage since: (1) our study focused on the role of Gal-3 in regulating RANKL expression and osteoclastogenesis; (2) RANKL is expressed at very low levels in BMM; (3) 1α,25(OH)_2_D_3_ increased Gal-3 expression only weakly in BMM (2-fold) but dramatically in BMSC (>10-fold). In addition, the transfection efficiency is extremely low in macrophages, and it is technically challenging to conduct experiments in macrophages.

Next, the regulatory role of Gal-3 in the expression of RANKL was demonstrated in BMSCs. The results confirmed that the expression of *RANKL* mRNA was downregulated by knockdown of Gal-3, and the expression of RANKL mRNA and the ratio of RANKL/OPG were upregulated by overexpression of Gal-3. The current results are different from those of Simon et al., who reported that inhibition of osteoclastogenesis was regulated by Gal-3 in mice independently of the RANKL/OPG axis. This might be related to differences in species variation or the different degrees of knockdown or overexpression of Gal-3 used.

**Figure 7:**
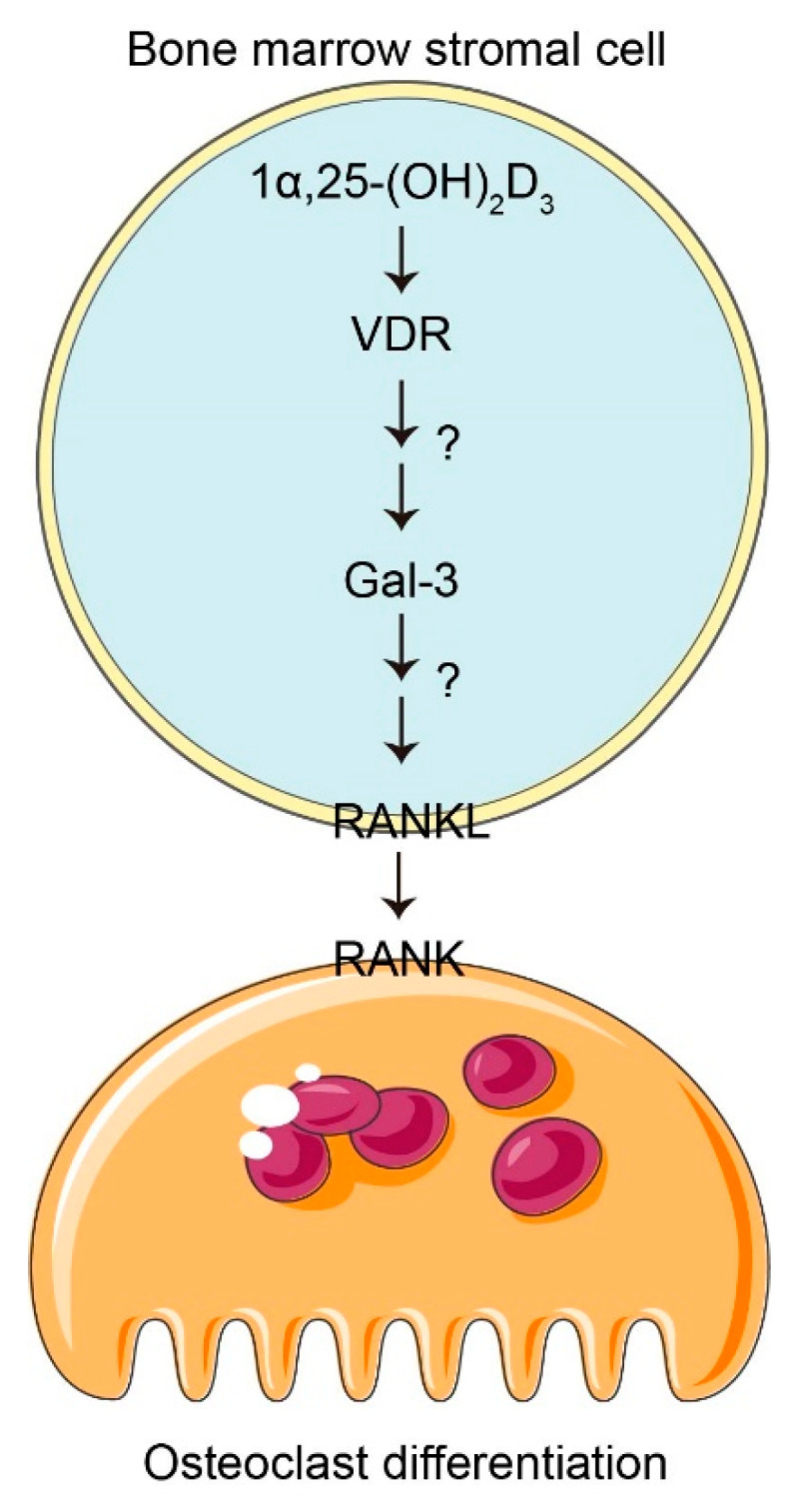
Schematic diagram showing the mechanism of Gal-3 in 1α,25(OH)_2_D_3_-regulated differentiation of chicken osteoclasts. Application of 10^−8^ mol/L 1α,25(OH)_2_D_3_ upregulates Gal-3 expression in BMSC and regulates osteoclast differentiation in the chicken through RANKL signaling.

Our present study shows that 1α,25-(OH)_2_D_3_ increased the levels of *Gal-3* and *RNAKL* mRNA and promoted osteoclast differentiation and activation. Gal-3 knockdown led to decreased *RANKL* mRNA expression and blocked the effect of 1α,25-(OH)_2_D_3_ on osteoclast differentiation. Gal-3 overexpression increased *RANKL* mRNA levels. It is well documented that Gal-3 present in the nucleus and can bind several transcription factors and enhance their DNA-binding activity [31]. We propose that 1α,25(OH)_2_D_3_-induced Gal-3 expression affects osteoclast differentitation through RANKL signaling in chicken BMSC (Figure 7). However, the mechanisms by which 1α,25-(OH)_2_D_3_ regulates Gal-3 expression and Gal-3 regulates RANKL expression need to be further investigated in detail.

## Figures and Tables

**Table 1:** Sequences of target genes.

Name	Gene Number	Length (bp)	Forward Primer (5′ to 3′)	Reverse Primer (5′ to 3′)
*CtsK* (chicken) *en)*	395818	161	CATCATTGACGGAGCGATGC	TTTCGTCCTCCTTGCCGTTG
*TRAP* (chicken)	107057619	110	CCGCTTCTTCTATGGGGCT	GGTACAGAACTCTCCCGTTGG
*MMP-9* (chicken)	395387	182	CTTCTGCCCAGACAGAGGTT	AGCCACGACCATAGAGGTACT
*CAII* (chicken)	396257	188	GGCGTGAAGTACGATGCAGA	GCTTGTTTCCCCTTGGTTTGAA
*RANKL* (chicken)	428067	116	GCCCACTTCTTGGAAACAGC	TACAACGTGGCCCTGTGAAG
*OPG* (chicken)	378803	90	CTGCACGCTTGTGCTCTTGG	GATGTCCCCGGGTCGTAATG
*Galectin-3* (chicken)	373917	112	GCCATATCCTGGAGGACCAAC	GGTTATGAGCAGTCGAGGCA
*GAPDH* (chicken)	374193	137	GCCCAGAACATCATCCCAG	CGGCAGGTCAGGTCAACA

## Data Availability

The raw data supporting the conclusions of this article will be made available by the authors.
